# Colorectal Cancer Treatment Delay Thresholds and Metastasis Risk

**DOI:** 10.1001/jamanetworkopen.2026.23057

**Published:** 2026-07-14

**Authors:** Chi M. Nguyen, Todd C. Skaar, Thomas F. Imperiale, Travis S. Johnson, Patrick O. Monahan, Anita A. Turk, Mai P. Nguyen

**Affiliations:** 1 Department of Biostatistics & Health Data Science, Indiana University School of Medicine, Indianapolis; 2 Division of Clinical Pharmacology, Department of Medicine, Indiana University School of Medicine, Indianapolis; 3 Division of Gastroenterology and Hepatology, Department of Medicine, Indiana University School of Medicine, Indianapolis; 4 Division of Hematology/Oncology, Department of Medicine, Indiana University School of Medicine, Indianapolis; 5 Asia Pacific Research Center, Freeman Spogli Institute for International Studies, Stanford University, Stanford, California

## Abstract

**Importance:**

Timely treatment in colorectal cancer (CRC) may influence the disease course and, thus, outcomes, but optimal delay thresholds remain uncertain. Identifying pathway-specific time-to-treatment initiation (TTI) effects can guide benchmarks and policies for coordinated, timely care.

**Objective:**

To evaluate the association between TTI and 3-year metastasis risk in patients with newly diagnosed nonmetastatic CRC undergoing curative-intent surgery, with attention to variation by treatment pathway.

**Design, Setting, and Participants:**

This cohort study of insured US patients with nonmetastatic CRC used deidentified administrative claims from Optum’s Clinformatics Data Mart. Eligible individuals were adults aged 40 years or older with incident nonmetastatic CRC diagnosed during January 1, 2017, to December 31, 2021, who underwent curative-intent surgery within 1 year of diagnosis and had continuous coverage 1 year before and after. Data were analyzed June 2025.

**Exposures:**

TTI was defined as days from CRC diagnosis to the first receipt of cancer-directed therapy (surgery, chemotherapy, or radiation). Patients were categorized into 4 treatment pathways: surgery with or without radiation, surgery followed by adjuvant therapy with or without radiation, neoadjuvant therapy followed by surgery with or without radiation, and trimodality therapy (neoadjuvant therapy, followed by surgery, followed by adjuvant therapy with or without radiation).

**Main Outcomes and Measures:**

The outcome was 3-year cumulative incidence of metastasis. XGBoost identified optimal TTI thresholds, and Fine-Gray models with death as a competing risk evaluated these thresholds and estimated their associations with metastasis.

**Results:**

Among 11 927 patients (mean [SD] age, 70.7 [10.8] years; 6007 women [50.4%]; 1251 Black [10.5%], 7948 White [66.6%]), 4539 (38.0%) had moderate or severe comorbidity. Over 3 years, 1438 patients (12.1%) developed metastasis. Longer TTIs were associated with higher metastasis risk, varying by treatment pathway. For surgery plus adjuvant therapy, TTIs of 4 to 46 days (subdistribution hazard ratio [sHR], 1.27; 95% CI, 1.04-1.55) and 47 days or longer (sHR, 1.55; 95% CI, 1.08-2.23) were significantly associated with increased risk vs zero to 3 days. For surgery followed by radiation, delays of 223 days or longer showed higher risk (sHR, 2.00; 95% CI, 0.95-4.25) than TTI up to 222 days, although these results were not significant. For neoadjuvant therapy plus surgery, TTI of 68 days or longer were associated with higher risk (sHR, 2.66; 95% CI, 1.02-6.94) than TTI up to 67 days. For patients receiving trimodality therapy, there was no association between TTI and risk of metastasis.

**Conclusions and Relevance:**

In this cohort study, treatment delays were associated with higher, pathway-specific metastasis risk. These findings support pathway-tailored benchmarks for treatment initiation and highlight the importance of integrated care in reducing delays and improving timely, equitable, cost-effective CRC care.

## Key Points

**Question:**

Are delays in time-to-treatment initiation (TTI) associated with increased metastatic progression risk in patients with newly diagnosed nonmetastatic colorectal cancer (CRC), and do these risks differ across treatment pathways?

**Findings:**

In this cohort study of 11 927 insured US patients with nonmetastatic CRC, treatment delays were associated with higher 3-year metastasis risk, with thresholds varying by treatment pathway. Even modest delays of 4 days or more were associated with higher metastatic risk in patients receiving surgery and adjuvant chemotherapy. Conversely, for surgery-only cases, only prolonged postponements (223 days or longer) indicated elevated risk, although this correlation lacked statistical significance.

**Meaning:**

These results suggest that pathway-specific thresholds for treatment initiation may be more effective than a single universal threshold in improving outcomes, promoting timely, cost-effective CRC care, and guiding clinical practice and performance benchmarks.

## Teaser

This cohort study of insured US patients with colorectal cancer undergoing curative-intent surgery examines the association of delays in treatment with metastasis.

## Introduction

Timely treatment initiation is critical in colorectal cancer (CRC) management, particularly for patients with nonmetastatic stage (non-mCRC). Delays may permit progression to metastasis, which is associated with more complex care, greater risk of toxic effects, diminished quality of life, and substantially higher costs.^1,2,3,4,5,6,7,8,9^ From a health care systems perspective, metastatic CRC (mCRC) has been shown to impose more expenditure than early-stage disease, with cumulative costs rising sharply over time,^10,11,12,13,14,15^ underscoring the importance of timely treatment to improve outcomes and reduce burden.

Prior studies of treatment timing in CRC mostly examine overall survival or system-level barriers, often using universal delay cutoffs.^16,17,18^ Although guidelines provide stage-specific therapeutic recommendations,^19,20^ they offer little guidance on acceptable treatment delays across care pathways. In practice, treatment sequencing varies substantially, ranging from surgery-first approaches to neoadjuvant-based regimens, and the clinical implications of delay may differ accordingly. Whether there are pathway-specific thresholds beyond which delay is associated with increased metastatic risk is unknown. This study addresses the gap by evaluating the association between time-to-treatment initiation (TTI) and 3-year metastasis risk among patients newly diagnosed with non-mCRC, aiming to inform evidence-based benchmarks for timely, cost-effective CRC care.

## Methods

### Study Design and Data

We conducted a cohort study of patients with newly diagnosed non-mCRC undergoing curative-intent surgery, using Optum’s Clinformatics Data Mart (CDM), licensed by Indiana University.^21,22^ CDM, the largest national US claims database, contains more than 75 million commercial and Medicare Advantage members (between 2007 and 2024), including enrollment, diagnoses, procedures, and prescriptions. Because this study used deidentified, HIPAA-compliant data.^23^ it was exempt from institutional review board review per the Common Rule. This study followed Strengthening the Reporting of Observational Studies in Epidemiology (STROBE) reporting guidelines.

The cohort included patients aged 40 years or older with incident non-mCRC diagnosed between January 2017 and December 31, 2021, undergoing curative-intent surgery within 1 year. This ensured that disease identification used *International Classification of Diseases, Tenth Revision, Clinical Modification* (*ICD-10-CM*) guidelines effective in November 2015, and ensured up to 3 years of follow-up (through December 2024).

**Figure 1.**
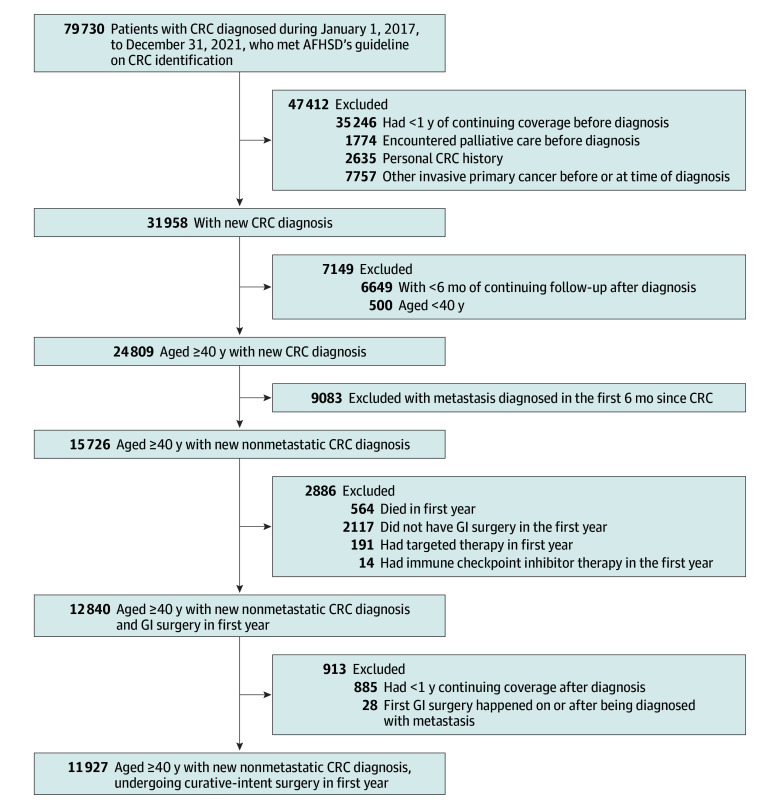
Derivation of Patients With Newly Diagnosed Nonmetastatic Colorectal Cancer (CRC) and Undergoing Curative-Intent Surgery in the First Year Guidelines derived from the US Armed Forces Health Surveillance Division (AFHSD) case definitions.^29^ GI indicates gastrointestinal.

Patients were additionally required to have: (1) at least 1 year of continuous insurance coverage before diagnosis; (2) no prior invasive cancer; (3) no evidence of metastasis within 6 months after diagnosis; (4) continuous coverage and survival at least 1 year after diagnosis; (5) surgery before metastatic progression; and (6) no receipt of targeted or immune checkpoint inhibitors in the first year, as these are Food and Drug Administration (FDA)-approved first-line metastatic treatments for biomarker-defined subgroups.^24,25,26,27,28^ Figure 1 illustrates cohort derivation.

### Nonmetastatic CRC and Metastasis Identification

Diagnoses were identified using *ICD-10-CM* codes. Following the US Armed Forces Health Surveillance Division guidelines on CRC case definition and incidence rules, codes C18.x, C19.x, C20, and C26.0 with treatment-encounter codes Z51.0 and Z51.1 defined new CRC cases and classified tumor location (colon, rectum, or both), with the first encounter date specified as the incident date.^30^ To ensure inclusion of only nonmetastatic CRC, patients needed at least 6 months of continuous follow-up (continuous coverage and at least 1 postdiagnosis record) without replacement of CRC codes by a metastasis code. Metastases were identified using *ICD-10-CM* codes for secondary malignant neoplasms of lymph nodes and organs (eTable 1 in Supplement 1). Coding algorithms were based on established, previously validated methods in administrative data.^30,31,32^

### Primary Outcome

The endpoint was the 3-year cumulative incidence of metastasis from the date of treatment initiation, defined as the patient’s first receipt of any cancer-directed therapy, including gastrointestinal surgery, chemotherapy, or radiation. Follow-up continued until the earliest of documented metastasis, death, or 3 years of observation; patients who disenrolled or were lost to follow-up before 3 years without mCRC were censored on their last observable date.

### TTI, Treatment Pathways, and Other Covariates

TTI was defined as the number of days from the first CRC diagnosis date to the date of treatment initiation. Cancer-directed treatments were identified through insurance claims using Current Procedural Terminology (CPT) and Healthcare Common Procedure Coding System (HCPCS) procedure codes, supplemented by the CDM Type of Service (TOS) classification (eTable 2 in Supplement 1). Operative digestive system TOS categories captured gastrointestinal surgical procedures, including colonoscopy, endoscopy, proctoscopy, laparoscopy, lesion excision, and colectomy or colorectal resection. Chemotherapy was identified through TOS drug administration categories and pharmacy claims for antineoplastic agents and regimens including fluorouracil, leucovorin, capecitabine, cisplatin, oxaliplatin, and irinotecan, or their combinations such as FOLFOX, FOLFIRI, FOLFOXIRI, XELOX, and XELIRI. Radiation therapy was identified using radiologic delivery procedures.

Based on treatment sequence in the first year, patients were classified into 4 pathways: (1) surgery with or without radiation; (2) neoadjuvant therapy followed by surgery with or without radiation; (3) surgery followed by adjuvant therapy with or without radiation; and (4) neoadjuvant therapy, followed by surgery, followed by adjuvant therapy with or without radiation (hereafter the *trimodality* pathway). As first-line therapy usually follows stage-specific guidelines,^33,34^ these pathways serve as pragmatic proxies for baseline disease invasiveness when stage is not reported (eTable 3 in Supplement 1).

Additional variables included demographics (sex, race or ethnicity as reported in CDM’s enrollment database, age) and preexisting comorbidities. Comorbidities were identified from all diagnoses prior to CRC diagnosis. Charlson Comorbidity Index (CCI) was calculated using the HCUP Charlson Comorbidity Software for *ICD-10-CM*^35,36^ and categorized as: none (CCI of 0), mild (1 to 2), moderate (3 to 4), and severe (5 or higher).

### Statistical Analysis

Baseline characteristics were summarized using frequencies for categorical variables and means or medians for continuous variables. A 2-stage, data-driven approach identified treatment-delay thresholds, defined as the TTI values at which metastasis risk increased. In stage 1, extreme gradient boosted regression trees (XGBoost)^37^ with a Cox model and TTI as the sole feature identified optimal thresholds differentiating metastasis risk. To capture nonlinear effects, tree depth (maximum number of thresholds allowed) ranged from 2 to 10, with 1000 boosting rounds per model (learning rate 0.1 to prevent overfitting^37^). The 3 highest-quality cutoffs per model were retained, and dominant thresholds were defined as those most frequently identified within 7-day sliding windows.

In stage 2, thresholds were tested in full sample using Fine-Gray models where all-cause death without metastasis was a competing risk. Adjacent TTI intervals with similar effects were grouped to improve interpretability and stability. Pathway-specific associations were estimated as subdistribution hazard ratios (sHR) with 95% CIs, adjusting for sex, race or ethnicity, age, CCI, and radiation. Sensitivity analyses using the diagnosis date as the index date assessed robustness of the estimated associations conditioning on treatment initiation. Analyses were conducted in R version 4.1.2 (R Project for Statistical Computing), with 2-sided *P* < .05 indicating a statistically significant result.

## Results

### Characteristics of the Study Cohort

Among 11 927 patients with newly diagnosed non-mCRC undergoing curative-intent surgery, the mean (SD) age at diagnosis was 70.7 (10.8) years; 6007 patients (50.4%) were women, and 1251 were Black (10.5%) and 7948 White (66.6%) (Table 1). A total of 1438 patients (38.0%) had moderate to severe comorbidity (ie, CCI of 3 or higher). Colon tumors accounted for 7869 cases (66.0%), rectal tumors 1257 cases (10.5%), and combined colon-rectal tumors 2801 cases (23.5%). Patients had the mean (SD) TTI of 11.6 (30.5) days, with a mean (SD) follow-up of 944 (257) days. Overall, 1438 patients (12.1%) developed metastasis and 806 (6.8%) died without metastasis within 3 years.

**Table 1.** Cohort Characteristics, Time-to-Treatment Initiation, and Follow-Up by Treatment Pathway^a^

Variable	Patients, No. (%)				
	Surgery (n = 9329)	Neoadjuvant therapy + surgery (n = 239)	Surgery + adjuvant therapy (n = 2175)	Neoadjuvant, surgery, and adjuvant (n = 184)	Total (N = 11 927)
Diagnosis age, y					
Mean (SD)	71.7 (10.4)	69.1 (11.5)	67 (11.2)	63.7 (11.7)	70.7 (10.8)
Median (IQR)	72.6 (66.7-79.7)	70.2 (61.4-78.5)	69.1 (58.5-75)	66.6 (53.2-73.3)	71.8 (65.4-78.8)
Diagnosis age group, y					
40-49	304 (3.3)	19 (7.9)	200 (9.2)	24 (13.0)	547 (4.6)
50-64	1704 (18.3)	56 (23.4)	566 (26.0)	63 (34.2)	2389 (20.0)
65-74	3538 (37.9)	79 (33.1)	865 (39.8)	61 (33.2)	4543 (38.1)
≥75	3783 (40.6)	85 (35.6)	544 (25.0)	36 (19.6)	4448 (37.3)
Sex					
Female	4846 (51.9)	113 (47.3)	980 (45.1)	68 (37.0)	6007 (50.4)
Male	4483 (48.1)	126 (52.7)	1195 (54.9)	116 (63.0)	5920 (49.6)
Race or ethnicity					
Asian	257 (2.8)	12 (5.0)	77 (3.5)	7 (3.8)	353 (3.0)
Black	985 (10.6)	19 (7.9)	233 (10.7)	14 (7.6)	1251 (10.5)
Hispanic	410 (4.4)	13 (5.4)	135 (6.2)	17 (9.2)	575 (4.8)
White	6369 (68.3)	152 (63.6)	1324 (60.9)	103 (56.0)	7948 (66.6)
Unknown	1308 (14.0)	43 (18.0)	406 (18.7)	43 (23.4)	1800 (15.1)
CCI score, No. (%)					
0	2220 (23.8)	76 (31.8)	744 (34.2)	69 (37.5)	3109 (26.1)
1-2	3337 (35.8)	85 (35.6)	779 (35.8)	78 (42.4)	4279 (35.9)
3-4	1923 (20.6)	41 (17.2)	380 (17.5)	17 (9.2)	2361 (19.8)
≥5	1849 (19.8)	37 (15.5)	272 (12.5)	20 (10.9)	2178 (18.2)
Tumor location in first 6 mos					
Colon	6974 (74.8)	24 (10.0)	867 (39.9)	<5 (2.2)	7869 (66.0)
Rectum	632 (6.8)	103 (43.1)	459 (21.1)	63 (34.2)	1257 (10.5)
Colon and rectum	1723 (18.5)	112 (46.9)	849 (39.0)	117 (63.6)	2801 (23.5)
Radiation in first year	154 (1.7)	201 (84.1)	966 (44.4)	173 (94.0)	1494 (12.5)
Time to treatment initiation, d					
Mean (SD)	11.7 (32.9)	31.0 (24.2)	7.3 (17.5)	29.4 (17.1)	11.6 (30.5)
Median (IQR)	0 (0-7)	27 (17-41)	0 (0-4)	27 (18-35)	0 (0-9)
Realized follow-up time, d					
Mean (SD)	964 (238.8)	886.6 (293)	870.1 (304.8)	862.8 (312.6)	943.7 (257.3)
Median (IQR)	1095 (934-1095)	1095 (721-1095)	1095 (616-1095)	1095 (612.5-1095)	1095 (854-1095)
Follow-up status					
Metastasis	795 (8.5)	53 (22.2)	546 (25.1)	44 (23.9)	1438 (12.1)
All-cause death before metastasis	685 (7.3)	13 (5.4)	100 (4.6)	8 (4.4)	806 (6.8)

Abbreviation: CCI, Charlson Comorbidity Index.

^a^ All characteristics were statistically significantly different at *P* < .001 for the omnibus test comparing 4 treatment pathways.

These characteristics differed across pathways. Male individuals were more prevalent in chemotherapy-inclusive regimens than female (116 of 184 [63.0%] in trimodality with or without radiation vs 4483 of 9329 [48.1%] in surgery only). Higher proportions of Hispanic and unknown race or ethnicity in chemotherapy groups. Severe preexisting comorbidity (CCI of 5 or higher) was less prevalent in the trimodality than in the surgery group (20 of 184 [10.9%] vs 1849 of 9329 [19.8%]), while the proportion with no comorbidities was higher (69 of 184 [37.5%] vs 2220 of 9329 [23.8%]). Patients receiving more intensive treatments were generally younger, with mean (SD) age ranging from 63.7 (11.7) years in the trimodality to 71.7 (10.4) years in the surgery group.

Regarding tumor sites, colon cases accounted for 74.8% (6974 of 9329 cases) in the surgery group yet was only 2.2% (fewer than 5 of 184 cases) in the trimodality group, whereas both colon-rectum cancers comprised 63.6% (117 of 184 cases) of the trimodality group. First-year radiation was utilized in 154 of 184 cases (1.7%) with surgery alone, rising to 966 of 2175 cases (44.4%) with surgery and adjuvant therapy, 201 of 239 cases (84.1%) with neoadjuvant therapy and surgery, and peaking at 173 of 184 (94.0%) in the trimodality group.

TTIs also varied considerably across pathways. Surgery-first pathways had a median TTI of zero days, with most patients receiving either colonoscopy with lesion removal or colectomy within zero to 3 days (eTable 4 in Supplement 1). Neoadjuvant-based pathways had longer, structured delays, with median TTIs of approximately 27 days and most patients initiating neoadjuvant therapy within 3 to 6 weeks. The overall median TTI of zero days was driven by the predominance of the surgery pathway (78.2%).

Meanwhile, the surgery pathway had the longest mean (SD) follow-up (964 [239] days) while other pathways ranged from 863 (313) to 887 (293) days. Incident metastases were lowest for surgery alone (8.5%) and highest for surgery with adjuvant therapy (25.1%), with trimodality (23.9%) and neoadjuvant-surgery (22.2%) intermediate (eTable 5 in Supplement 1).

### Data-Driven TTI Thresholds

**Figure 2.**
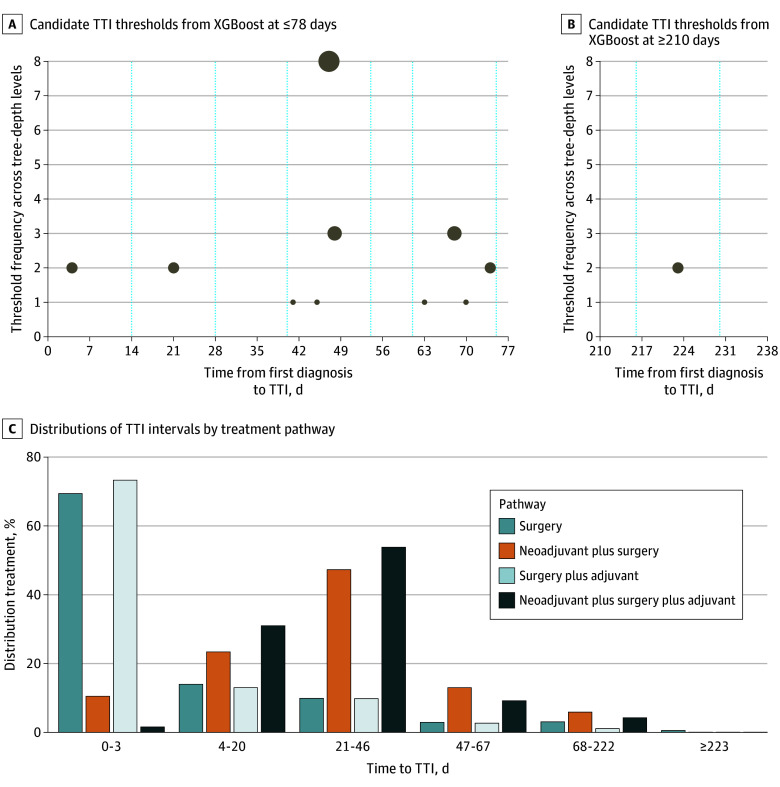
Time-to-Treatment Initiation (TTI) Thresholds and Their Distributions by Treatment Pathway The size of plotted circles indicate the threshold frequency across tree-depth level.

The 5 TTI thresholds most frequently occurring across XGBoost models were 4, 21, 47, 68, and 223 days resulting in 6 intervals (zero to 3 days, 4 to 20 days, 21 to 46 days, 47 to 67 days, 68 to 222 days, and 223 days and longer (Figure 2A; eTable 6 in Supplement 1). Most patients in the surgery-only (69.3%) and surgery and adjuvant therapy groups (73.3%) initiated treatment within 3 days. Neoadjuvant-based pathways had longer delays: 47.3% of neoadjuvant and surgery and 53.8% of trimodality patients started treatment between 21 and 46 days. TTIs beyond 46 days were also more common in these groups (Figure 2B).

### TTI Thresholds’ Associations With Metastasis

When evaluating the TTI intervals in full sample, results suggested that even seemingly short delays may negatively affect outcomes (eTable 7 in Supplement 1). Particularly, compared with treatment within 3 days, TTI of 4 to 20 days (sHR, 1.19; 95% CI, 1.03-1.38) and 21 to 46 days (sHR, 1.18; 95% CI, 1.00-1.39) were both significantly associated with approximately 18% to 19% higher 3-year cumulative metastasis incidence.

Meanwhile, pathway-specific evaluation of these intervals revealed more directions (eTable 8 in Supplement 1). In the surgery group, TTI of 223 days or longer had higher metastasis risk (sHR, 2.05; 95% CI, 0.97-4.35), although this result was not statistically significant. Results were not significant in the neoadjuvant plus surgery group, where shorter (4 to 20 days: sHR, 0.48; 95% CI, 0.06-3.83) and moderate delays (47 to 67 days: sHR, 0.66; 95% CI, 0.09-4.77) had lower HRs than past 68 days (sHR, 1.70; 95% CI, 0.23-12.84). In the surgery plus adjuvant group, delays came with higher risk, with 47 to 67 days reaching statistical significance (sHR, 1.55; 95% CI, 1.00-2.38), while hazard ratios in the trimodality group varied by interval.

**Figure 3.**
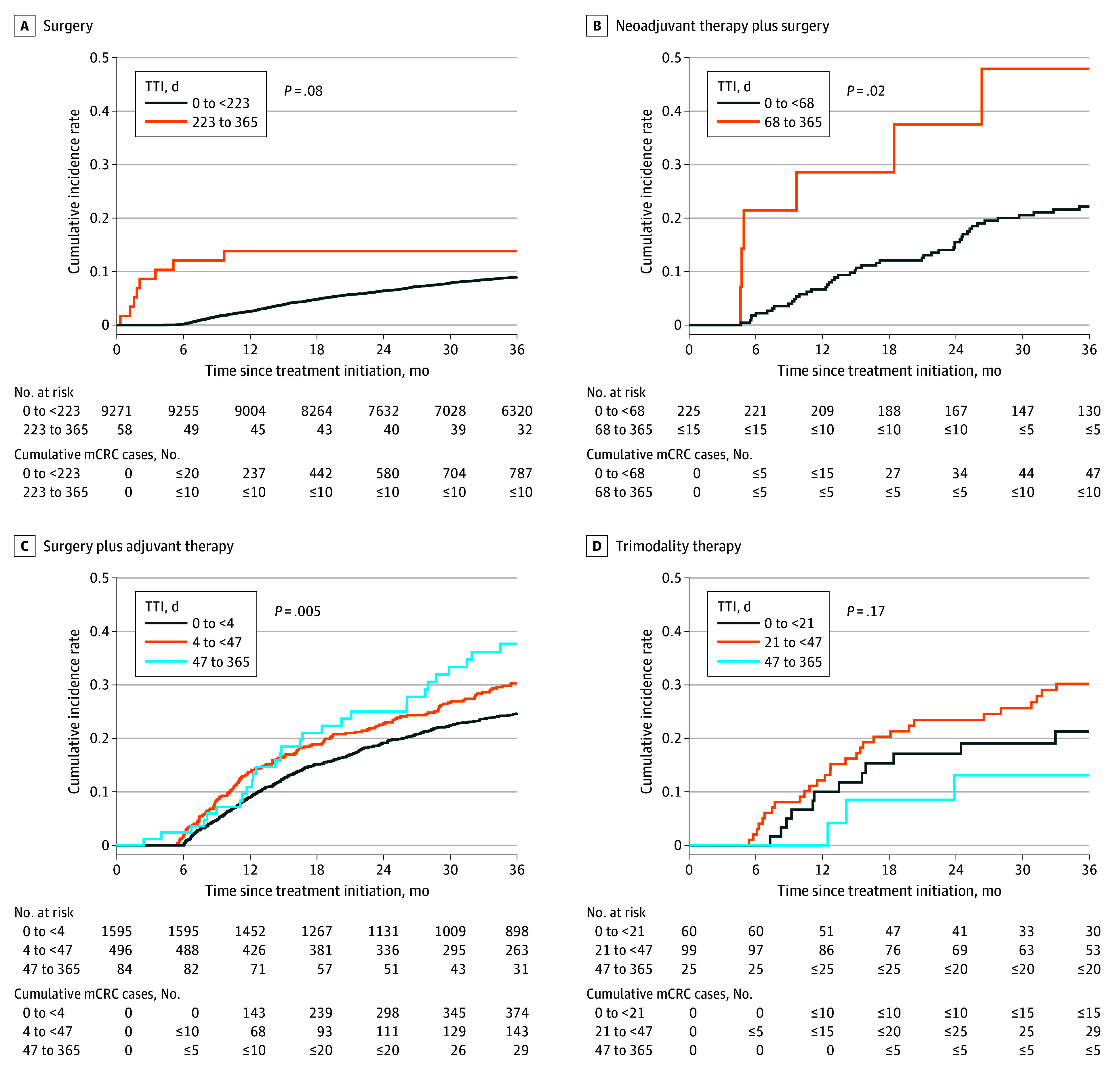
Three-Year Cumulative Incidence of Metastasis by Time-to-Treatment Initiation (TTI) Interval, With Death as a Competing Risk Aalen-Johansen cumulative incidence of metastasis by TTI group (accounting for death as a competing risk), with Gray *P* values testing for group differences. Per the Optum CDM Data User Agreement, counts 25 or below were reported in increments of 5 (≤5, ≤10, ≤15, ≤20, and ≤25). mCRC indicates metastatic colorectal cancer.

After merging adjacent TTI categories into broader, directionally consistent intervals, pathway-specific comparisons were refined. In the surgery-only pathway, TTIs less than 223 days were compared with 223 days or more. For neoadjuvant therapy plus surgery, TTIs were dichotomized at 68 days, where risk increased sharply. In surgery plus adjuvant therapy, TTIs 4 days or later were grouped as 4 to 46 days and 47 days or more, reflecting higher risk beyond 6 weeks. In the trimodality pathway, TTIs were grouped into 0 to 20, 21 to 46, and 47 days or more to reflect stepwise timing effects. Figure 3 displays the unadjusted 3-year cumulative metastasis incidence across these intervals.

Adjusted associations for the refined TTI intervals were shown in Table 2, with results for demographics and comorbidities in eTable 9 of Supplement 1. In the surgery group (74.8% with colon cancer), TTI of 223 days or longer showed nearly twice the cumulative risk vs shorter intervals, although results did not reach statistical significance (sHR, 2.00; 95% CI, 0.95-4.25; *P* = .07). Tumor location within 6 months showed no significant association. Radiation within the first year was associated with metastasis, with a large effect size (sHR, 2.85; 95% CI, 2.00-4.07; *P* < .001).

**Table 2.** Adjusted Associations of the Refined TTI Intervals With 3-Year Cumulative Incidence of Metastasis

Characteristic	sHR (95% CI)	*P* value
**Surgery**		
TTI ≥223 d (vs 0-222 d)	2.00 (0.95-4.25)	.07
Tumor location in the first 6 mo		
Colon	1 [Reference]	[Reference]
Rectum	1.03 (0.78-1.36)	.82
Colon and rectum	1.12 (0.94-1.34)	.21
Had radiation in the first year	2.85 (2.00-4.07)	<.001
**Neoadjuvant therapy + surgery**		
TTI ≥68 d (vs 0-67 d)	2.66 (1.02-6.94)	.046
Tumor location in the first 6 mo		
Colon^a^	1 [Reference]	[Reference]
Rectum	9.19 (0.96-87.71)	.05
Colon and rectum	9.90 (1.09-89.58)	.04
Had radiation in the first year	0.79 (0.32-1.95)	.60
**Surgery + adjuvant therapy**		
TTI 0-3 d	1 [Reference]	[Reference]
TTI 4-46 d	1.27 (1.04-1.55)	.02
TTI ≥47 d	1.55 (1.08-2.23)	.02
Tumor location in the first 6 mo		
Colon	1 [Reference]	[Reference]
Rectum	1.14 (0.80-1.61)	.48
Colon and rectum	1.08 (0.84-1.39)	.53
Had radiation in the first year	1.03 (0.79-1.35)	.82
**Neoadjuvant therapy + surgery + adjuvant therapy**		
TTI 0-20 d	1 [Reference]	[Reference]
TTI 21-46 d	1.60 (0.80-3.18)	.18
TTI ≥47 d	0.58 (0.17-1.96)	.38
Tumor location in the 1st 6 mo		
Colon^b^	1 [Reference]	[Reference]
Rectum	1.61 (0.1-26.23)	.74
Colon and rectum	1.94 (0.13-30.0)	.64
Had radiation in the first year	0.47 (0.07-3.32)	.45

Abbreviations: sHR, subdistribution hazard ratio; TTI, time-to-treatment initiation.

^a^ Colon cancer had ≤25 cases in this pathway.

^b^ Colon cancer had ≤5 cases in this pathway.

In the neoadjuvant therapy and surgery group (90% rectal or both colon-rectal cancer), TTI of 68 days or longer was significantly associated with higher risk (sHR, 2.66; 95% CI, 1.02-6.94; *P* = .046). Tumors involving both sites also showed significantly higher cumulative risk vs colon only (sHR, 9.90; 95% CI, 1.09-89.58; *P* = .04). Tumors in the rectum also showed higher cumulative risk, although results were not significant (sHR, 9.19; 95% CI, 0.96-87.71; *P* = .05). Radiation therapy was not significantly associated with metastasis.

In the surgery and adjuvant therapy group (nearly 60% rectal or both colon-rectal cancers), treatment timing was statistically significant on metastasis. Compared with patients who initiated treatment within 3 days, those with TTIs of 4 to 46 days had a 27% higher cumulative risk (sHR, 1.27; 95% CI, 1.04-1.55; *P* = .02), and those with TTIs 47 days or longer had a 55% higher risk (sHR, 1.55; 95% CI, 1.08-2.23; *P* = .02), reflecting the sensitivity of the outcomes to TTIs within the first 6 weeks. Neither tumor location nor radiation exposure were significant. Finally, in the trimodality pathway (nearly 98% rectal or combined colon-rectal cancer), neither significant timing effects nor tumor location or radiation associations were observed.

### Sensitivity Analysis

Analysis revealed that findings were generally robust to diagnosis date as the index, with consistent pathway-specific patterns showing longer TTI associated with higher metastasis risk (eTable 10 in Supplement 1). Notably, delays remained significantly associated with the risk in the surgery plus adjuvant pathway, were borderline in the neoadjuvant plus surgery pathway (longer than 68 days), and were not significant in the trimodality pathway, reinforcing the pathway-dependent timing effects. Associations of other covariates were also consistent with the primary analysis (eTable 11 in Supplement 1).

## Discussion

TTI varies considerably across treatment pathways. Shorter TTIs are common for surgery-first approaches, whereas longer TTIs are expected in neoadjuvant-based regimens, reflecting guideline-concordant sequencing rather than inefficiency.^17,18^ When delays arise from administrative or systemic barriers rather than medical necessity, they have been linked to worse outcomes across multiple cancers, including CRC.^1,2,18,38^ In this large clinical cohort, the prognostic relevance of TTI was pathway dependent, supporting pathway-specific rather than universal thresholds.

In the surgery-only pathway, delays exceeding 7 months (223 days or longer) showed a higher 3-year cumulative metastasis risk, although results were not significant. This small subgroup was predominantly colon cancer and included older patients with higher comorbidity burden than the overall cohort (eTables 5 and 12 in Supplement 1). While brief postponements may reflect necessary evaluation or scheduling, prolonged delays may signal care fragmentation or delayed engagement, potentially permitting tumor progression.^38^ Although not statistically significant, this finding supports monitoring prolonged delays, especially in early-stage patients where curative resection is definitive. Radiation was significantly associated with metastasis only in this pathway, likely reflecting more aggressive tumors and served as a marker of higher underlying risk.

Among patients receiving neoadjuvant therapy followed by surgery, TTIs of 68 days or longer (ie, more than 9 weeks) were significantly associated with higher risk. Because this pathway typically represents patients with locally advanced or large tumors, such delays may reflect nonclinical barriers, such as prior authorization, insurance appeals, or scheduling bottlenecks.^39,40^ Additional contributors may include transportation challenges, comorbidity management, and financial hardship.^41^ Given the sophisticated biology often present in this group, delays beyond 10 weeks may undermine therapeutic benefit, particularly in biologically aggressive tumors.

The strongest timing effect sizes were observed in patients undergoing surgery followed by adjuvant chemotherapy. Delays of 4 to 46 days were associated with increased metastasis risk, and delays beyond 47 days with a 55% increase. The very short thresholds likely reflect clinical care sequencing rather than a literal biological deadline, as same-day colonoscopy procedures are common; small delays may therefore distinguish streamlined from fragmented care. Contrarily, delays beyond 6 or 7 weeks likely represent a practical inflection point, potentially allowing biologically active tumors additional time to disseminate in higher-risk patients, aligning with established tumor biology, as deeper invasion increases the likelihood of lymphovascular spread and metastatic competence.^42,43,44,45^

Not all early delays are clinically avoidable as short TTIs often encompass necessary surgery scheduling, preoperative evaluation, and multidisciplinary coordination.^46^ Emerging minimally invasive biomarkers, such as multitarget stool DNA testing^47^ and circulating tumor DNA assays,^47,48^ may help identify when tumor biology transitions and detect what could be described as molecular “tipping points” toward systemic dissemination, to guide prioritization when metastatic potential is imminent.

In contrast, TTI was not significantly associated with higher risk in the trimodality pathway. These patients often had locally advanced disease requiring multidisciplinary coordination among surgical, medical, and radiation oncologists,^40^ where longer TTIs are often planned and medically appropriate. Moreover, sequential systemic therapy before and after surgery can suppress micrometastatic disease, potentially offsetting delay effects.^8,9,18,19^ Nevertheless, unplanned interruptions or excessive deferrals within multimodal regimens can still compromise outcomes and warrant monitoring.^38^

Overall, very short TTIs, including zeros, were more common in our cohort than in previous studies^49^ (median [IQR], 0 [0-9] days vs 11 [0-30] days), likely due to differences in study periods, cohort compositions, and claims-based data structure. In our cohort, 78.2% of patients underwent surgery alone, reflecting early-stage management. The short TTI likely reflects streamlined care coordination in insured populations, high colonoscopy uptake as an initial surgical intervention, the predominance of the surgery-first pathways, and limitations inherent to claims-based capture where preceding diagnostic workup may have occurred outside the observable claims window. Accordingly, zero TTI should be interpreted cautiously as indicating prompt observable care following incident CRC, rather than same-day definitive treatment. Future studies with more granular clinical sequencing data should further evaluate the interval from diagnosis to definitive treatment.

This study offers 3 contributions. First, it examined metastasis incidence rather than overall survival, providing a more direct measure of disease control. Second, it identified empirically derived, machine learning–informed, pathway-specific thresholds rather than arbitrary cutoffs, enhancing clinical relevance. Third, it leveraged a large, nationally representative cohort of 11 927 insured patients with longitudinal treatment data, enabling robust evaluation of TTI and informing clinical and policy translation.

Our findings suggest implications for care delivery and policy development, although they should be interpreted cautiously given its observational and claims-based nature. Pathway-specific TTI thresholds may help inform future performance measures and resource allocation, particularly for surgery plus adjuvant chemotherapy where delays beyond 6 weeks may be hypothetically associated with higher metastasis risk. Potential strategies to reduce avoidable delays could include parallel consultations, streamlined pathology workflows, patient navigation, multidisciplinary coordination, and expedited insurance authorization processes.^38,40,41^ Because mCRC imposes greater clinical and economic burden than early-stage disease,^10,11,12,13,14,15^ preventing metastasis through timely treatment aligns with value-based care and sustainability.

### Limitations

Our study had several limitations. Claims data lack detailed tumor characteristics (stage, grade, depth of invasion, molecular profile), and limit to claims-based capture, leaving potential TTI misclassification and unmeasured residual confounding despite pathway stratification. Findings should, therefore, be interpreted as associative rather than causal. The cohort, primarily of commercially insured and Medicare Advantage beneficiaries, may limit generalizability to underserved populations. Unmeasured factors, including patient preference and institutional capacity, may influence TTI and metastasis risk. Future research integrating clinical registry and genomic data, with further characterization of procedural sequencing, could refine prognostic stratification and validate whether pathway-specific thresholds could supplement existing quality metrics and support value-based care in broader populations.

## Conclusions

In this cohort study of patients with non-mCRC undergoing curative-intent surgery, treatment delays were associated with higher metastasis risk in a pathway-dependent manner. Six to seven weeks of delays were consequential for patients requiring surgery and adjuvant chemotherapy. These findings underscore the importance of coordinated, timely care and support the development of pathway-specific quality benchmarks and integrated-care models to minimize avoidable delays. Ensuring prompt access to CRC treatment remains an evidence-based priority for improving patient outcomes and advancing equitable, cost-effective care.

## Notes

Supplement 1. eTable 1. Diagnosis codes for primary invasive colorectal cancer and metastasis cancers eTable 2. Identification of cancer treatments by types of medical facility, services and procedure codes provided by Optum CDM eTable 3. Pathway explanations with guideline links eTable 4. Distribution of gastrointestinal surgical procedure types, procedure frequency, and most common CPT code combinations in surgery-first pathways eTable 5. Summary of metastasis cases by pathway and baseline tumor location eTable 6. TTI candidate cutoffs identified at different tree depth eTable 7. Adjusted associations of TTI thresholds with metastasis risk in full sample eTable 8. Adjusted association of TTIs with metastasis risk in each treatment pathway eTable 9. Effects of other factors associated with the mCRC risk, after regrouping TTI intervals (Treatment Initiation-based index date) eTable 10. Adjusted association of TTI with the cumulative metastasis risk by treatment pathway (diagnosis-based index date) eTable 11. Effects of other factors associated with the metastasis risk (diagnosis-based index date) eTable 12. Baseline description of patients with prolonged TTI ≥223 days in the surgery pathway

Supplement 2. Data Sharing Statement
