# Supplementary material for: Colorectal Cancer Treatment Delay Thresholds and Metastasis Risk
Source: JAMA Netw Open. 2026 Jul 14;9(7):e2623057. doi: 10.1001/jamanetworkopen.2026.23057 (PMC13370307; doi:10.1001/jamanetworkopen.2026.23057)
Supplement: Supplement 1. — eTable 1. Diagnosis codes for primary invasive colorectal cancer and metastasis cancers eTable 2. Identification of cancer treatments by types of medical facility, services and procedure codes provided by Optum CDM eTable 3. Pathway explanations with guideline links eTable 4. Distribution of gastrointestinal surgical procedure types, procedure frequency, and most common CPT code combinations in surgery-first pathways eTable 5. Summary of metastasis cases by pathway and baseline tumor location eTable 6. TTI candidate cutoffs identified at different tree depth eTable 7. Adjusted associations of TTI thresholds with metastasis risk in full sample eTable 8. Adjusted association of TTIs with metastasis risk in each treatment pathway eTable 9. Effects of other factors associated with the mCRC risk, after regrouping TTI intervals (Treatment Initiation-based index date) eTable 10. Adjusted association of TTI with the cumulative metastasis risk by treatment pathway (diagnosis-based index date) eTable 11. Effects of other factors associated with the metastasis risk (diagnosis-based index date) eTable 12. Baseline description of patients with prolonged TTI ≥223 days in the surgery pathway [file jamanetwopen-e2623057-s001.pdf]

Supplemental Online Content

Nguyen CM, Skaar TC, Imperiale TF, et al. Colorectal cancer treatment delay thresholds and metastasis risk. *JAMA Netw Open.* 2026;9(7):e2623057. doi:10.1001/jamanetworkopen.2026.23057

eTable 1. Diagnosis codes for primary invasive colorectal cancer and metastasis cancers

eTable 2. Identification of cancer treatments by types of medical facility, services and procedure codes provided by Optum CDM

eTable 3. Pathway explanations with guideline links

eTable 4. Distribution of gastrointestinal surgical procedure types, procedure frequency, and most common CPT code combinations in surgery-first pathways

eTable 5. Summary of metastasis cases by pathway and baseline tumor location

eTable 6. TTI candidate cutoffs identified at different tree depth

eTable 7. Adjusted associations of TTI thresholds with metastasis risk in full sample

eTable 8. Adjusted association of TTIs with metastasis risk in each treatment pathway

eTable 9. Effects of other factors associated with the mCRC risk, after regrouping TTI intervals (Treatment Initiation-based index date)

eTable 10. Adjusted association of TTI with the cumulative metastasis risk by treatment pathway (diagnosis-based index date)

eTable 11. Effects of other factors associated with the metastasis risk (diagnosis-based index date)

eTable 12. Baseline description of patients with prolonged TTI ≥223 days in the surgery pathway

This supplemental material has been provided by the authors to give readers additional information about their work.

© 2026 Nguyen et al. *JAMA Network Open*.

eTable 1: Diagnosis codes for primary invasive colorectal cancer and metastasis cancers

ICD-10	Description	Source
Primary invasive colorectal cancer		
C18	Malignant neoplasm of colon	Case identification and Incident rules of colorectal cancer by AFHSD
C19	Malignant neoplasm of rectosigmoid junction	
C20	Malignant neoplasm of rectum	
C26.0	Malignant neoplasm of intestinal tract, part unspecified	
Metastasis cancer		
C77.x	Secondary malignant neoplasm of lymph nodes	ICD-10-CM and HCUP
C78.0	Secondary malignant neoplasm of lung	
C78.1	Secondary malignant neoplasm of mediastinum	
C78.2	Secondary malignant neoplasm of pleura	
C78.3	Secondary malignant neoplasm of respiratory organ	
C78.4	Secondary malignant neoplasm of small intestine	
C78.5	Secondary malignant neoplasm of large intestine and rectum	
C78.6	Secondary malignant neoplasm of retroperitoneum and peritoneum	
C78.7	Secondary malignant neoplasm of liver and intrahepatic bile duct	
C78.8	Secondary malignant neoplasm of other or unspecified digestive organ	
C79.0	Secondary malignant neoplasm of kidney and renal pelvis	
C79.1	Secondary malignant neoplasm of urinary organs	
C79.2	Secondary malignant neoplasm of skin	
C79.3	Secondary malignant neoplasm of brain, cerebral meninges	
C79.4	Secondary malignant neoplasm of unspecified or other part of nervous system	
C79.5	Secondary malignant neoplasm of bone	
C79.6	Secondary malignant neoplasm of ovary	
C79.7	Secondary malignant neoplasm of adrenal gland	
C79.81	Secondary malignant neoplasm of breast	
C79.82	Secondary malignant neoplasm of genital organs	
C79.89	Secondary malignant neoplasm of other specified sites	
C79.9	Secondary malignant neoplasm of unspecified site	
C7B.00	Secondary carcinoid tumors, unspecified site	
C7B.01	Secondary carcinoid tumors of distant lymph nodes	
C7B.02	Secondary carcinoid tumors of liver	
C7B.03	Secondary carcinoid tumors of bone	
C7B.04	Secondary carcinoid tumors of peritoneum	
C7B.09	Secondary carcinoid tumors of other sites	

© 2026 Nguyen et al. *JAMA Network Open*.

C7B.1	Secondary Merkel cell carcinoma
C7B.8	Other secondary neuroendocrine tumors
C80.0	Disseminated malignant neoplasm, unspecified

© 2026 Nguyen et al. *JAMA Network Open*.

eTable 2: Identification of cancer treatments by types of medical facility, services and procedure codes provided by Optum CDM

Type of Service – Treatment: Specialty	Description	Type-of-Service in detail
**GI surgery[(a)]**		
Facility outpatient - Surgery: Digestive	Colonoscopy surgeries, Abdomen or peritoneum or omentum, anus, appendix, biliary tract, esophagus, intestines, liver, meckel and mesentery, pancreas, rectum, stomach	FAC_OP.FO_SUR.DIGEST.CLNSCP, FAC_OP.FO_SUR.AMBSRG.ABDPER, FAC_OP.FO_SUR.DIGEST.ABDPER, FAC_OP.FO_SUR.AMBSRG.ABDOM, FAC_OP.FO_SUR.DIGEST.ABDOM, FAC_OP.FO_SUR.AMBSRG.ANUS, FAC_OP.FO_SUR.DIGEST.ANUS, FAC_OP.FO_SUR.AMBSRG.APPEND, FAC_OP.FO_SUR.DIGEST.APPEND, FAC_OP.FO_SUR.AMBSRG.BILIAR, FAC_OP.FO_SUR.DIGEST.BILIAR, FAC_OP.FO_SUR.AMBSRG.ESOPH, FAC_OP.FO_SUR.DIGEST.ESOPH, FAC_OP.FO_SUR.AMBSRG.INTEST, FAC_OP.FO_SUR.DIGEST.INTEST, FAC_OP.FO_SUR.AMBSRG.LIVER, FAC_OP.FO_SUR.DIGEST.LIVER, FAC_OP.FO_SUR.AMBSRG.MECK, FAC_OP.FO_SUR.DIGEST.MECK, FAC_OP.FO_SUR.AMBSRG.PANCR, FAC_OP.FO_SUR.DIGEST.PANCR, FAC_OP.FO_SUR.AMBSRG.RECT, FAC_OP.FO_SUR.DIGEST.RECT, FAC_OP.FO_SUR.AMBSRG.STOM, FAC_OP.FO_SUR.DIGEST.STOM
Professional services - Surgery: Digestive	Abdomen/peritoneum/omentum, anus, appendix, biliary tract, esophagus, intestines, liver, meckel and mesentery, pancreas, rectum, stomach	PROF.SURG.DIGEST.ABDPER, PROF.SURG.DIGEST.ANUS, PROF.SURG.DIGEST.APPEND, PROF.SURG.DIGEST.BILIAR, PROF.SURG.DIGEST.ESOPH, PROF.SURG.DIGEST.INTEST, PROF.SURG.DIGEST.LIVER, PROF.SURG.DIGEST.MECK, PROF.SURG.DIGEST.PANCR, PROF.SURG.DIGEST.RECT, PROF.SURG.DIGEST.STOM
**Cancer Pharmacotherapies[(b)]**		

© 2026 Nguyen et al. *JAMA Network Open*.

Ancillary - Drugs administered: Antineoplastic agents	Alkylating agents, nitrogen mustards; anthracycline antibiotics; antiestrogens, chemotherapeutic; antimetabolites; antineoplastic antibiotics, miscellaneou; antineoplastic radiopharmaceuticals; antineoplastics, miscellaneous; chemotherapy adjuncts; cytoprotective agents, chemotherapeutic; gonadotropin releasing hormones, chemoth; mitotic inhibitors, podophyllotoxin deri; mitotic inhibitors, taxanes; mitotic inhibitors, vinca alkaloids; topoisomerase inhibitors	ANC.DRUGAD.6602, excluding targeted therapy (e.g., bevacizumab, cetuximab, panitumumab, aflibercept, trifluridine and tipiracil), and Immune Checkpoint Inhibitor immunotherapy (e.g., nivolumab, pembrolizumab, atezolizumab, cemiplimab, ipilimumab, avelumab, and durvalumab)
Facility outpatient - Other: Cancer therapy	Chemotherapy	FAC_OP.FO_OTH.CHEMO
Professional services - Professional other: Cancer therapy	Chemotherapy	PROF.PROOTH.CHEMO
**Radiation therapy[c]**		
Facility outpatient - Radiology: Therapeutic radiology	Brachytherapy; radionuclide therapy; therapeutic radiology; treatment delivery; treatment management; treatment planning	FAC_OP.FO_RAD.THRRAD.BRACHY, FAC_OP.FO_RAD.THRRAD.NUCLID, FAC_OP.FO_RAD.THRRAD.THRRAD, FAC_OP.FO_RAD.THRRAD.TRTDEL, FAC_OP.FO_RAD.THRRAD.TRTMGT, FAC_OP.FO_RAD.THRRAD.TRTPLN
Professional services - Radiology: Therapeutic radiology	Brachytherapy; nuclear medicine; other; radiation oncology; radionuclide therapy; treatment delivery; treatment management; treatment planning	PROF.RAD.THRRAD.BRACHY, PROF.RAD.THRRAD.NUCMED, PROF.RAD.THRRAD.OTHER, PROF.RAD.THRRAD.RADONC, PROF.RAD.THRRAD.NUCLID, PROF.RAD.THRRAD.TRTDEL, PROF.RAD.THRRAD.TRTMGT, PROF.RAD.THRRAD.TRTPLN

Notes: These types of services were linked with Current Procedural Terminology (CPT-4) and Healthcare Common Procedure Coding System (HCPCS) as follows.

[a] *Gastrointestinal surgery* associated with CPT-4/HCPCS procedure codes, including S2083, C9779, 0437T, 0355T, 0184T, 49999, 49906, 49905, 49904, 49900, 49659, 49657, 49656, 49655, 49654, 49653, 49652, 49651, 49650, 49622, 49621, 49596, 49595, 49594, 49593, 49592, 49591, 49590, 49587, 49585, 49572, 49570, 49568, 49566, 49565, 49561, 49560, 49553, 49550, 49525, 49521, 49520, 49507, 49505, 49465, 49460, 49452, 49451, 49450, 49446, 49441, 49440, 49436, 49429, 49424, 49423, 49422, 49421, 49419, 49418, 49412, 49407, 49406, 49405, 49402, 49400, 49329, 49327, 49326, 49325, 49324, 49323, 49322, 49321, 49320, 49255, 49250, 49215, 49205, 49204, 49203, 49185, 49180, 49084, 49083, 49082, 49060, 49040, 49020, 49010, 49002, 49000, 48999, 48153, 48150, 48148, 48140, 48120, 48105, 48102, 47999, 47801, 47785, 47780, 47760, 47715, 47711, 47700, 47610, 47605, 47600, 47579, 47564, 47563, 47562, 47554, 47550, 47540, 47538, 47537, 47536, 47535, 47534, 47533, 47532, 47531, 47490, 47480, 47420, 47399, 47383, 47382, 47381, 47380, 47379, 47370, 47362, 47350, 47300, 47143, 47135, 47130, 47125, 47122, 47120, 47100, 47015, 47010, 47001, 47000, 46999, 46947, 46946, 46945, 46940, 46930, 46924, 46922, 46910, 46900, 46750, 46710, 46706, 46615, 46614, 46612, 46611, 46610, 46608, 46607, 46606, 46604, 46601, 46600, 46505, 46500, 46320, 46288, 46280, 46275, 46270, 46260, 46255, 46250, 46230, 46221, 46220, 46200, 46083, 46080, 46060, 46050, 46040, 46030, 46020, 45999, 45990, 45915,

© 2026 Nguyen et al. *JAMA Network Open*.

45910, 45905, 45900, 45805, 45800, 45562, 45560, 45550, 45541, 45520, 45505, 45500, 45499, 45400, 45399, 45398, 45397, 45395, 45393, 45392, 45391, 45390, 45389, 45388, 45386, 45385, 45384, 45382, 45381, 45380, 45379, 45378, 45350, 45349, 45347, 45346, 45342, 45341, 45340, 45338, 45337, 45335, 45334, 45333, 45332, 45331, 45330, 45327, 45320, 45317, 45315, 45309, 45308, 45307, 45305, 45303, 45300, 45190, 45172, 45171, 45160, 45150, 45135, 45130, 45126, 45123, 45121, 45120, 45119, 45114, 45113, 45112, 45111, 45110, 45100, 45020, 45005, 45000, 44979, 44970, 44960, 44955, 44950, 44900, 44899, 44850, 44820, 44800, 44799, 44705, 44701, 44700, 44661, 44660, 44650, 44640, 44626, 44625, 44620, 44615, 44605, 44604, 44603, 44602, 44500, 44408, 44405, 44404, 44403, 44401, 44394, 44392, 44391, 44389, 44388, 44386, 44385, 44382, 44381, 44380, 44379, 44378, 44377, 44376, 44373, 44369, 44366, 44364, 44363, 44361, 44360, 44346, 44345, 44340, 44320, 44316, 44314, 44312, 44310, 44300, 44238, 44227, 44213, 44212, 44211, 44210, 44208, 44207, 44206, 44205, 44204, 44203, 44202, 44188, 44187, 44186, 44180, 44160, 44158, 44157, 44156, 44155, 44151, 44150, 44147, 44146, 44145, 44144, 44143, 44141, 44140, 44139, 44130, 44125, 44121, 44120, 44111, 44110, 44100, 44055, 44050, 44025, 44021, 44020, 44015, 44010, 44005, 43999, 43870, 43860, 43848, 43840, 43830, 43820, 43800, 43775, 43774, 43762, 43761, 43760, 43753, 43752, 43659, 43653, 43644, 43640, 43633, 43632, 43631, 43621, 43611, 43610, 43605, 43499, 43453, 43450, 43332, 43327, 43310, 43289, 43287, 43285, 43282, 43281, 43280, 43279, 43278, 43277, 43276, 43275, 43274, 43273, 43270, 43266, 43265, 43264, 43262, 43261, 43260, 43259, 43255, 43254, 43253, 43252, 43251, 43250, 43249, 43248, 43247, 43246, 43245, 43244, 43243, 43242, 43241, 43240, 43239, 43238, 43237, 43236, 43235, 43233, 43232, 43231, 43229, 43227, 43226, 43220, 43213, 43212, 43202, 43200, 43197, 43196, 43191, 43180, 43130, 43117, 22999, 22905, 22903, 22901, 22900.

[(b)] Chemotherapies for colorectal cancer: single regiment or different combinations of fluorouracil, leucovorin, capecitabine, cisplatin, oxaliplatin, and irinotecan. These drug administrations were associated with procedure codes including S9331, S9330, S9329, Q2050, Q0084, J9999, J9400, J9395, J9370, J9360, J9358, J9356, J9355, J9351, J9309, J9308, J9306, J9305, J9303, J9298, J9293, J9280, J9267, J9264, J9263, J9260, J9250, J9245, J9229, J9223, J9217, J9216, J9209, J9208, J9206, J9205, J9201, J9200, J9198, J9190, J9185, J9181, J9171, J9130, J9100, J9070, J9060, J9045, J9044, J9041, J9040, J9039, J9034, J9031, J9030, J9025, J9000, J8610, J8521, J8520, J3315, J2783, J1950, J1190, J0894, J0594, G0498, G0070, A9543, A9529, A9517, 96549, 96542, 96450, 96446, 96425, 96422, 96420, 96417, 96416, 96415, 96413, 96411, 96409, 96406, 96405, 96402, 96401, 96367, 81002.

[(c)] *Radiation* associated with procedure codes including Q3001, G6017, G6016, G6015, G6014, G6013, G6012, G6011, G6009, G6005, G6002, G6001, G0340, G0339, C9728, 77799, 77790, 77778, 77772, 77771, 77770, 77620, 77615, 77610, 77605, 77600, 77525, 77523, 77522, 77470, 77469, 77435, 77432, 77431, 77427, 77424, 77423, 77417, 77412, 77407, 77402, 77401, 77399, 77387, 77386, 77385, 77373, 77372, 77371, 77370, 77338, 77336, 77334, 77333, 77332, 77331, 77321, 77318, 77317, 77316, 77307, 77306, 77301, 77300, 77299, 77295, 77290, 77285, 77280, 77263, 77262, 77261, 77014, 74283, 49411, 32701.

Abbreviations for Type of Service:
FAC_OP or FO – Facility outpatient; PROF – Professional service
SUR or SURG – Surgery; AMBSRG – Ambulatory Surgery
CLNSCP – Colonoscopy
DIGEST – Digestive System:
ABDPER – Abdomen/peritoneum/omentum ; ABDOM – Abdomen ; ANUS – Anus; APPEND – Appendix; BILIAR – biliary tract ; ESOPH – esophagus ; INTEST – Intestines ; MECK – Meckel and mesentery; PANCR – Pancreas ; RECT – Rectum ; STOM – Stomach
ANC.DRUGAD.6602 – Ancillary: DRUgs Administered: Antineoplastic agents
CHEMO – Chemotherapy
RAD.THRRAD – RADiology: THRerapeutic RADiology
BRACHY – Brachytherapy; NUCMED – nuclear medicine; NUCLID – radionuclide therapy; RADONC – radiation oncology; TRTDEL – treatment delivery; TRTMGT – treatment management; TRTPLN – treatment planning

© 2026 Nguyen et al. *JAMA Network Open*.

eTable 3: Pathway explanations with guideline links

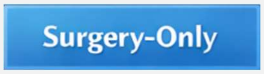

Patients in the surgery-only pathway typically have early-stage, screen-detected colorectal cancer, for which definitive surgical resection is the primary curative treatment and is often performed on the day of diagnosis or shortly thereafter.

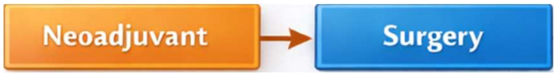

Neoadjuvant therapy is intentionally initiated before surgery to downsize locally advanced tumors, most commonly rectal cancer, allowing treatment to begin promptly after diagnosis while definitive resection is deliberately deferred as part of guideline-concordant sequencing rather than due to care inefficiency. Although neoadjuvant therapy is not definitive treatment, it represents critical early cancer-directed care that improves tumor control, and resectability as emphasized in contemporary NCCN rectal cancer guidelines.

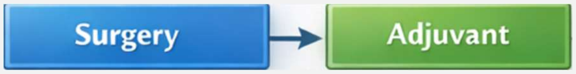

In the adjuvant pathway, surgery constitutes definitive treatment, followed by chemotherapy after postoperative recovery to reduce recurrence risk. Adjuvant therapy is usually for stage III and selected high-risk stage II.

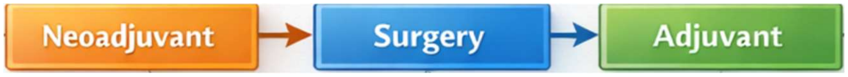

(Tri-modality)

Tri-modality treatment reflects locally advanced disease requiring planned sequencing of neoadjuvant therapy, definitive surgery, and postoperative chemotherapy. It is usually for rectal or both colorectal cancer stage III.

Guidelines:

- **National Comprehensive Cancer Network (NCCN) Colon Cancer Guidelines** https://www.nccn.org/guidelines/guidelines-detail?category=1&id=1428
- **National Comprehensive Cancer Network (NCCN) Rectal Cancer Guidelines** https://www.nccn.org/guidelines/guidelines-detail?category=1&id=1461

© 2026 Nguyen et al. *JAMA Network Open*.

eTable 4: Distribution of Gastrointestinal Surgical Procedure Types, Procedure Frequency, and Most Common CPT Code Combinations in surgery-first pathways

GI surgery procedure type	# of persons (%)	# of GI procedures: Median (IQR)	Most frequently combined CPT codes and their description
**Surgery-only pathway**			
colonoscopy	5147 (55.2%)	2 (2 - 3)	45380 and/or 45381 with 45385: Colonoscopy with biopsy and/or submucosal injection, with colonoscopic lesion removal (e.g., snare polypectomy)
colectomy	3672 (39.4%)	1 (1 - 1)	either 44204 or 44205 or 44160: Partial colectomy or segmental resection of the colon (open or laparoscopic approach)
proctoscopy	242 (2.6%)	2 (1 - 2)	45331 and/or 45335 with 45338: Sigmoidoscopy with biopsy and/or submucosal injection, with endoscopic removal of tumor or lesion.
endoscopy	104 (1.1%)	3 (2 - 4)	43239, 43251, 45380: Esophagogastroduodenoscopy (EGD) with biopsy and/or endoscopic lesion removal (snare technique), and colonoscopy with biopsy.
laparoscopy	88 (0.9%)	1 (1 - 2)	44205, 49329: Laparoscopic partial colectomy (including ileum, when applicable) and unlisted laparoscopic abdominal, peritoneal, or omental procedure.
other operative therapeutic procedure	72 (0.8%)	2 (2 - 3)	0184T, 44625, 44626: Transanal endoscopic microsurgical excision of rectal tumor and repair or closure of bowel openings.

© 2026 Nguyen et al. *JAMA Network Open*.

lesion excision	≤5 (0%)	2 (1.75 - 2.25)	44110, 44205: Excision of intestinal lesion(s) and laparoscopic partial colectomy (including ileum, when applicable).
**Surgery + Adjuvant therapy pathway**			
colonoscopy	1351 (62.1%)	2 (2 - 3)	45380 and/or 45381 with 45385: Colonoscopy with biopsy and/or submucosal injection, with colonoscopic lesion removal (e.g., snare polypectomy)
colectomy	628 (28.9%)	1 (1 - 2)	either 44204 or 44205 or 44160: Partial colectomy or segmental resection of the colon (open or laparoscopic approach)
proctoscopy	146 (6.7%)	2 (1 - 2)	45331 and/or 45335 with 45338: Sigmoidoscopy with biopsy and/or submucosal injection, with endoscopic removal of tumor or lesion.
endoscopy	≤25 (1%)	3 (3 - 4)	43239, 43251, 45380: EGD with biopsy and/or endoscopic lesion removal (snare technique), and colonoscopy with biopsy.
laparoscopy	≤20 (0.9%)	2 (2 - 3)	44205, 49329: Laparoscopic partial colectomy (including ileum, when applicable) and unlisted laparoscopic abdominal, peritoneal, or omental procedure.
other operative therapeutic procedure	≤10 (0.4%)	2 (1 - 2)	0184T, 45172: Transanal excision of rectal tumor, including transanal endoscopic microsurgical approach and full-thickness local excision of rectal tumor.

© 2026 Nguyen et al. *JAMA Network Open*.

eTable 5: Summary of metastasis cases by pathway and baseline tumor location

Tumor location at baseline	TTI intervals (days)	# of persons	# of metastasis cases
**1. Surgery**			
Colon	[0,223)	6930	570
	[223,365)	44	≤10
Rectum	[0,223)	625	57
	[223,365)	≤10	0
Colon & rectum	[0,223)	1716	160
	[223,365)	≤10	≤5
**2. Neoadjuvant + Surgery**			
Colon	[0,68)	≤25	≤5
	[68,365)	≤5	0
Rectum	[0,68)	99	≤25
	[68,365)	≤5	≤5
Colon & rectum	[0,68)	103	≤25
	[68,365)	≤10	≤5
**3. Surgery + Adjuvant therapy**			
Colon	[0,4)	598	144
	[4,47)	232	57
	[47,365)	37	≤15
Rectum	[0,4)	375	91
	[4,47)	70	≤25
	[47,365)	≤15	≤5
Colon & rectum	[0,4)	622	139
	[4,47)	194	62
	[47,365)	33	≤15
**4. Tri-modality**			
Colon	[0,21)	≤5	≤5
	[21,47)	≤5	0
	[47,365)	0	0
Rectum	[0,21)	≤25	≤5
	[21,47)	31	≤10
	[47,365)	≤10	0
Colon & Rectum	[0,21)	36	≤10
	[21,47)	66	≤25
	[47,365)	≤15	≤5

© 2026 Nguyen et al. *JAMA Network Open*.

eTable 6: TTI candidate cutoffs identified at different tree depth

Tree depth	Quality score	Split points
2	7.36	45, 48
3	8.41	47, 48, 63
4	7.36	47, 48, 70
5	8.11	4, 21, 47
6	7.45	41, 47, 74
7	8.11	4, 21, 47
8	7.91	47, 68, 74
9	6.91	47, 68, 223
10	6.91	47, 68, 223

Note: In this study, tree depth corresponds to the maximum number of cutoff points allowed in each model

© 2026 Nguyen et al. *JAMA Network Open*.

eTable 7: Adjusted associations of TTI thresholds with metastasis risk in full sample

	Full sample N = 11,927	
Factor	sHR (95% CI)	P
**TTI category: Ref 0-3 days**		
4-20 days	1.19 (1.03, 1.38)	0.021
21-46 days	1.18 (1.00, 1.39)	0.047
47-67 days	1.13 (0.86, 1.50)	0.384
68-222 days	1.27 (0.93, 1.74)	0.127
≥223 days	1.84 (0.86, 3.94)	0.116
**Age group: Ref 40-49 years**		
[2] 50-64	1.03 (0.77, 1.36)	0.862
[3] 65-74	1.43 (1.09, 1.87)	0.009
[4] 75plus	1.44 (1.09, 1.90)	0.009
**Female**	0.88 (0.79, 0.97)	0.013
**Race/Ethnicity: Ref White**		
African	1.12 (0.95, 1.32)	0.195
Hispanic	0.73 (0.56, 0.95)	0.021
Asian	0.87 (0.64, 1.19)	0.395
Unknown	1.05 (0.90, 1.22)	0.564
**CCI score: Ref CCI = 0**		
1.Mild CCI 1-2	0.94 (0.82, 1.08)	0.413
2.Moderate CCI 3-4	1.03 (0.88, 1.21)	0.729
3.Severe CCI ≥5	1.10 (0.93, 1.30)	0.261
**Tumor location in 1st 6 months: Ref 1.Colon**		
2.Rectum	1.04 (0.85, 1.26)	0.719
3.Colon & rectum	1.10 (0.95, 1.26)	0.201
**Radiation in 1st year**	1.22 (1.00, 1.48)	0.048
**Treatment in 1s year: Ref Surgery**		
2. Neoadjuvant-Surgery	2.23 (1.62, 3.07)	<0.001
3. Surgery-Adjuvant	3.14 (2.73, 3.60)	<0.001
4. Neoadjuvant-Surgery-Adjuvant	2.46 (1.71, 3.52)	<0.001

© 2026 Nguyen et al. *JAMA Network Open*.

eTable 8: Adjusted association of TTIs with metastasis risk in each treatment pathway

	**1. Surgery N1 = 9,329**		**2. Neoadjuvant therapy + Surgery N2 = 239**		**3. Surgery + Adjuvant therapy N3 = 2,175**		**4. Neoadjuvant + Surgery + Adjuvant N4 = 184**	
	sHR (95% CI)	P	sHR (95% CI)	P	sHR (95% CI)	P	sHR (95% CI)	P
**TTI category: Ref 0-3 days**								
4-20 days	1.18 (0.97, 1.43)	0.089	0.48 (0.06, 3.83)	0.487	1.24 (0.97 ,1.59)	0.082	1.67 (0.02, 170.49)	0.827
21-46 days	0.96 (0.75, 1.23)	0.752	0.66 (0.09, 4.89)	0.688	1.31 (0.99 ,1.72)	0.057	2.60 (0.03, 236.53)	0.678
47-67 days	0.94 (0.61, 1.44)	0.762	0.66 (0.09, 4.77)	0.680	1.55 (1.00 ,2.38)	0.049	0.94 (0.01, 103.00)	0.980
68-222 days	1.06 (0.71, 1.58)	0.786	1.70 (0.23, 12.84)	0.606	1.57 (0.83 ,2.94)	0.164	0.93 (0.01, 129.48)	0.976
≥223 days	2.05 (0.97, 4.35)	0.062	NA		NA		NA	
**Diagnosis Age group: Ref 40-49 years**								
[2] 50-64	0.84 (0.52, 1.36)	0.476	1.95 (0.53, 7.21)	0.317	0.96 (0.66 ,1.40)	0.844	3.87 (0.67, 22.44)	0.131
[3] 65-74	1.19 (0.75, 1.88)	0.468	1.62 (0.43, 6.12)	0.476	1.48 (1.04 ,2.12)	0.031	3.81 (0.66, 21.93)	0.135
[4] 75plus	1.35 (0.85, 2.14)	0.209	1.57 (0.39, 6.37)	0.528	1.35 (0.85 ,1.83)	0.253	3.79 (0.51, 28.39)	0.195
**Female**	0.79 (0.69, 0.91)	0.001	0.96 (0.55, 1.69)	0.896	0.92 (0.81 ,1.14)	0.642	1.28 (0.67, 2.43)	0.452
**Race/Ethnicity: Ref White**								
African	1.16 (0.93, 1.44)	0.196	0.46 (0.11, 2.03)	0.307	1.11 (0.84 ,1.45)	0.467	1.41 (0.51, 3.94)	0.509
Hispanic	0.65 (0.43, 0.98)	0.040	0.62 (0.14, 2.68)	0.525	0.79 (0.54 ,1.17)	0.243	1.21 (0.35, 4.19)	0.762
Asian	0.81 (0.51, 1.29)	0.371	0.27 (0.04, 1.87)	0.184	0.94 (0.59 ,1.50)	0.791	1.81 (0.55, 5.95)	0.331
Unknown	0.90 (0.72, 1.13)	0.356	0.92 (0.46, 1.83)	0.819	1.22 (0.96 ,1.54)	0.105	1.10 (0.47, 2.60)	0.826
**CCI score: Ref CCI = 0**								
1. CCI 1-2	0.96 (0.79, 1.17)	0.698	1.04 (0.52, 2.09)	0.903	0.92 (0.74 ,1.15)	0.469	0.69 (0.34, 1.4)	0.305
2. CCI 3-4	0.98 (0.78, 1.22)	0.837	0.79 (0.30, 2.08)	0.630	1.13 (0.88 ,1.46)	0.346	0.40 (0.08, 2.13)	0.285
3. CCI ≥5	1.06 (0.85, 1.31)	0.629	1.12 (0.43, 2.9)	0.817	1.16 (0.88 ,1.53)	0.306	1.00 (0.31, 3.26)	0.995
**Tumor location in 1st 6 months: Ref 1.Colon**								
2.Rectum	1.03 (0.78, 1.36)	0.838	11.91 (1.1, 128.83)	0.041	1.14 (0.8 ,1.61)	0.474	2.01 (0.02, 162.29)	0.756
3.Colon & rectum	1.12 (0.94, 1.34)	0.205	13.02 (1.27, 133.29)	0.031	1.08 (0.84 ,1.39)	0.527	2.42 (0.03, 192.1)	0.692
**Radiation in 1st year**	2.87 (2.02, 4.10)	0.000	0.88 (0.3, 2.61)	0.816	1.03 (0.79 ,1.35)	0.814	0.37 (0.01, 10.34)	0.555

© 2026 Nguyen et al. *JAMA Network Open*.

eTable 9: Effects of other factors associated with the mCRC risk, after regrouping TTI intervals (Treatment Initiation-based index date)

	**1. Surgery**		**2. Neoadjuvant + Surgery**		**3. Surgery + Adjuvant**		**4. Neoadjuvant + Surgery + Adjuvant**	
	**sHR (95% CI)**	**P**	**sHR (95% CI)**	**P**	**sHR (95% CI)**	**P**	**sHR (95% CI)**	**P**
**Dx age group - Ref: [1] 40-lt50**								
[2] 50-64	0.84 (0.52, 1.36)	0.479	2.14 (0.6, 7.71)	0.243	0.96 (0.67, 1.4)	0.851	3.69 (0.76, 17.88)	0.105
[3] 65-74	1.19 (0.75, 1.88)	0.460	1.77 (0.49, 6.45)	0.387	1.48 (1.04, 2.12)	0.031	3.58 (0.74, 17.26)	0.112
[4] 75plus	1.36 (0.85, 2.16)	0.196	1.78 (0.47, 6.78)	0.401	1.25 (0.85, 1.84)	0.250	3.65 (0.55, 24.09)	0.178
**Female**	0.79 (0.69, 0.91)	0.001	0.99 (0.56, 1.73)	0.958	0.96 (0.81, 1.14)	0.648	1.29 (0.69, 2.4)	0.429
**Race/ethnicity - Ref: White**								
African	1.16 (0.93, 1.44)	0.186	0.45 (0.1, 1.97)	0.288	1.11 (0.85, 1.45)	0.455	1.35 (0.48, 3.8)	0.570
Hispanic	0.65 (0.43, 0.98)	0.038	0.64 (0.16, 2.6)	0.531	0.8 (0.54, 1.17)	0.243	1.20 (0.36, 3.99)	0.765
Asian	0.80 (0.5, 1.28)	0.353	0.29 (0.03, 2.57)	0.267	0.94 (0.59, 1.5)	0.790	1.80 (0.55, 5.87)	0.329
Unknown	0.90 (0.72, 1.13)	0.366	0.94 (0.48, 1.84)	0.864	1.22 (0.96, 1.54)	0.106	1.09 (0.47, 2.53)	0.848
**CCI - Ref: 0. CCI = 0**								
1. CCI 1-2	0.97 (0.8, 1.17)	0.721	1.06 (0.53, 2.1)	0.868	0.92 (0.74, 1.15)	0.465	0.68 (0.34, 1.38)	0.290
2. CCI 3-4	0.98 (0.79, 1.22)	0.862	0.83 (0.31, 2.19)	0.704	1.13 (0.88, 1.46)	0.345	0.43 (0.09, 2.08)	0.291
3. CCI 5+	1.06 (0.85, 1.32)	0.596	1.21 (0.48, 3.06)	0.691	1.15 (0.87, 1.52)	0.310	0.97 (0.3, 3.18)	0.958

© 2026 Nguyen et al. *JAMA Network Open*.

eTable 10: Adjusted association of TTI with the cumulative metastasis risk by treatment pathway (diagnosis-based index date)

	sHR (95% CI)	P
**1. Surgery**		
TTI ≥ 223 days (vs 0-222 days)	1.75 (0.84, 3.64)	0.134
Tumor location in the 1st 6 months		
1. Colon (reference)		
2. Rectum	1.03 (0.78, 1.36)	0.833
3. Colon and rectum	1.12 (0.94, 1.34)	0.214
Had radiation in the 1st year	2.85 (2, 4.06)	<0.001
**2. Neoadjuvant therapy + Surgery**		
TTI ≥ 68 days (vs 0-67 days)	2.49 (1.00, 6.22)	0.051
Tumor location in the 1st 6 months		
1. Colon (reference)		
2. Rectum	8.79 (0.92, 83.61)	0.059
3. Colon and rectum	9.55 (1.06, 85.94)	0.044
Had radiation in the 1st year	0.80 (0.33, 1.96)	0.626
**3. Surgery + Adjuvant therapy**		
TTI 0-3 days (reference)		
TTI 4-46 days	1.25 (1.02, 1.52)	0.032
TTI ≥ 47 days	1.45 (1.02, 2.07)	0.040
Tumor location in the 1st 6 months		
1. Colon (reference)		
2. Rectum	1.13 (0.8, 1.61)	0.480
3. Colon and rectum	1.08 (0.84, 1.39)	0.538
Had radiation in the 1st year	1.03 (0.79, 1.36)	0.804
**4. Neoadjuvant therapy + Surgery + Adjuvant therapy**		
TTI 0-20 days (reference)		
TTI 21-46 days	1.58 (0.79, 3.16)	0.193
TTI ≥ 47 days	0.56 (0.17, 1.89)	0.352
Tumor location in the 1st 6 months		
1. Colon (reference)(b)		
2. Rectum	1.58 (0.09, 26.41)	0.749
3. Colon and rectum	1.89 (0.12, 29.98)	0.651
Had radiation in the 1st year	1.58 (0.07, 3.3)	0.447

© 2026 Nguyen et al. *JAMA Network Open*.

eTable 11: Effects of other factors associated with the metastasis risk (diagnosis-based index date)

	1. Surgery		2. Neoadjuvant + Surgery		3. Surgery + Adjuvant		4. Neoadjuvant + Surgery + Adjuvant	
	**sHR (95% CI)**	**P**	**sHR (95% CI)**	**P**	**sHR (95% CI)**	**P**	**sHR (95% CI)**	**P**
**Dx age group - Ref: [1] 40-lt50**								
[2] 50-64	0.84 (0.52, 1.36)	0.482	2.13 (0.59, 7.65)	0.247	0.97 (0.67, 1.4)	0.853	3.69 (0.76, 17.89)	0.105
[3] 65-74	1.19 (0.75, 1.89)	0.455	1.78 (0.49, 6.45)	0.382	1.48 (1.04, 2.12)	0.030	3.57 (0.74, 17.17)	0.113
[4] 75plus	1.36 (0.86, 2.16)	0.194	1.77 (0.46, 6.74)	0.403	1.25 (0.85, 1.83)	0.251	3.63 (0.55, 23.95)	0.180
**Female**	0.79 (0.69, 0.91)	0.001	0.98 (0.56, 1.72)	0.956	0.96 (0.81, 1.14)	0.634	1.28 (0.69, 2.4)	0.432
**Race/ethnicity - Ref: White**								
African	1.16 (0.93, 1.44)	0.184	0.44 (0.1, 1.95)	0.281	1.11 (0.85, 1.45)	0.463	1.34 (0.48, 3.74)	0.582
Hispanic	0.65 (0.43, 0.97)	0.037	0.64 (0.16, 2.61)	0.529	0.8 (0.54, 1.17)	0.250	1.2 (0.36, 3.97)	0.769
Asian	0.8 (0.5, 1.27)	0.345	0.3 (0.03, 2.6)	0.273	0.94 (0.59, 1.5)	0.796	1.8 (0.55, 5.9)	0.331
Unknown	0.9 (0.72, 1.13)	0.368	0.94 (0.48, 1.83)	0.858	1.22 (0.96, 1.54)	0.105	1.08 (0.46, 2.53)	0.854
**CCI - Ref: 0. CCI = 0**								
1. CCI 1-2	0.97 (0.8, 1.17)	0.723	1.06 (0.53, 2.09)	0.873	0.92 (0.74, 1.15)	0.473	0.68 (0.34, 1.39)	0.291
2. CCI 3-4	0.98 (0.79, 1.22)	0.864	0.83 (0.31, 2.19)	0.703	1.13 (0.88, 1.45)	0.349	0.43 (0.09, 2.07)	0.290
3. CCI 5+	1.06 (0.85, 1.32)	0.601	1.18 (0.47, 2.97)	0.730	1.15 (0.88, 1.52)	0.309	0.97 (0.3, 3.21)	0.966

© 2026 Nguyen et al. *JAMA Network Open*.

eTable 12. Baseline description of patients with prolonged TTI ≥223 days in the surgery pathway

variable	value
**Total patients with TTI≥223**	58
**Gender**	
Female	33 (56.90%)
Male	25 (43.10%)
**Race/ethnicity**	
White	38 (65.52%)
African	≤10 (13.79%)
Hispanic	≤5 (3.45%)
Asian	0 (0%)
Unknown	≤10 (17.24%)
**Age at diagnosis**	
[1] 40-lt50	≤5 (3.45%)
[2] 50-lt65	≤15 (20.69%)
[3] 65-lt75	≤25 (43.10%)
[4] 75plus	≤20 (32.76%)
**Tumor location in the 1st 6 months of diagnosis**	
Colon	44 (75.86%)
Rectum	≤10 (12.07%)
Colon & Rectum	≤10 (12.07%)
**CCI score**	
CCI = 0	≤15 (20.69%)
CCI 1-2	≤25 (37.93%)
CCI 3-4	≤15 (20.69%)
CCI ≥ 5	≤15 (20.69%)

© 2026 Nguyen et al. *JAMA Network Open*.
