# Supplementary material for: Colorectal Cancer Treatment Delay Thresholds and Metastasis Risk
Source: JAMA Netw Open. 2026 Jul 14;9(7):e2623057. doi: 10.1001/jamanetworkopen.2026.23057 (PMC13370307; doi:10.1001/jamanetworkopen.2026.23057)
Supplement: Supplement 2. — Data Sharing Statement [file jamanetwopen-e2623057-s002.pdf]

Data Sharing Statement

Nguyen. Colorectal Cancer Treatment Delay Thresholds and Metastasis Risk. *JAMA Netw Open*. Published July 14, 2026. doi:10.1001/jamanetworkopen.2026.23057

Data
Data available: No

Additional Information
Explanation for why data not available: Optum's data is not sharable unless the entity interested in accessing the data obtains its own Data Use Agreement with Optum.
